# Nitrogen-containing carbon membrane-encapsulated MnO nanorods for ultrastable lithium-ion batteries

**DOI:** 10.1039/d6ra03883b

**Published:** 2026-07-13

**Authors:** QiuYun Yang, Jiaqiang Wei, Yumu Liu, Xiaoyu Fan, Chunjian Liu, Na Wang, Minglei Guo

**Affiliations:** a Institute of Electrical and Electronic Engineering, Anhui Science and Technology University Bengbu Anhui 233000 China yang1990@ustc.edu.cn

## Abstract

Among the transition metal oxides that have been studied for lithium-ion battery anodes, MnO is very attractive because it has good theoretical capacity, abundant raw materials, and low toxicity. Nevertheless, its practical performance is often limited by low electronic conductivity, the tendency of MnO particles to agglomerate, and substantial volume fluctuations during repeated lithiation/delithiation processes. These factors reduce reaction efficiency and gradually deteriorate cycling stability. To address the above problems, we put forward a simple two-step path: hydrothermal crystallisation and then polydopamine-assisted pyrolysis; it can be observed that MnO nanorods are formed, and these are then conformally covered with a nitrogen-rich carbon shell (N–C@MnO). A carbon shell can be used to provide mechanical flexibility and absorb the cyclic volume variation of the electrode, and at the same time, it has good electron conductivity. These synergistic structural features collectively contribute to the outstanding cycling stability and charge-storage properties delivered by the N–C@MnO electrode. The optimised N–C@MnO electrode delivers an initial discharge capacity of 1004.7 mA h g^−1^ and a high reversible capacity of 912.9 mA h g^−1^ subsequent to 100 cycles at 0.1 A g^−1^; and after 1400 cycles at a high rate of 5 A g^−1^, it is still 293.8 mA h g^−1^, demonstrating excellent structural durability under demanding operating conditions. The above results have provided a general basis for the structure of engineering high-energy-density conversion-type anodes, and this strategy offers a practical solution to improve the performance of next-generation lithium-ion batteries.

## Abstract 2

Nitrogen-doped carbon encapsulation constructs a conductive and robust interface on MnO, enabling accelerated electron/Li^+^ transport and enhanced structural stability for high-performance lithium-ion batteries.

## Graphical abstract

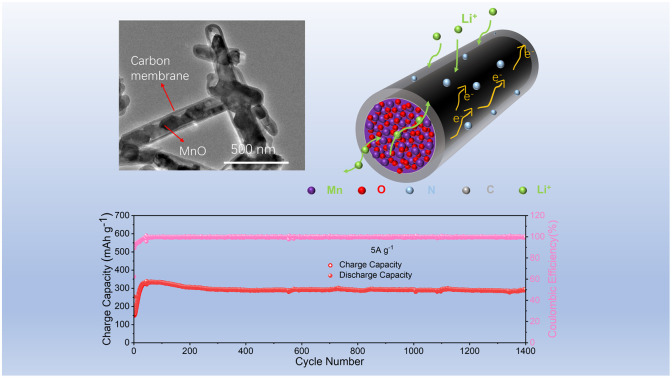

## Introduction

Manganese oxide has a relatively high theoretical charge storage capacity, but there are three persistent problems: poor intrinsic electronic conductivity, uncontrollable lattice swelling during lithiation and delithiation, and gradual pulverisation after many cycles; therefore, it has had limited application in practical LIB anodes, and both the capacity and service life of the electrode have been reduced.^1–4^ Based on this, various research has been conducted on remediation strategies that utilise nanostructuring, carbon hybridisation, intentional heteroatom substitution in the oxide lattice, *etc.*^5–17^ Cheng and his group have achieved some good results in the synthesis of hierarchical porous MnO/NC composites with flower-like and hollow-sphere structures; they have extended the extended cycle life at high rates significantly,^5^ and Ma *et al.* have prepared double-yolk–shell MnO/C microspheres by solvothermal annealing that still show good capacity retention after a long time.^10^ Some good results have also been shown in the engineering of heterojunctions; the Mn_2_O_3_/MnO@NC structure can form an internal electric field to promote both charge transfer and lithium-ion migration at the same time.^2,16^ Nitridation and multi-element co-doping have also increased the electron conductivity and structural stability of the surface.^14,17^ Taken together, the above research has demonstrated that a good route to enhance the performance of manganese oxide electrodes is to combine rational nanostructure design and carbonisation.

Although carbon-coated manganese oxides materials have been reported in recent years, it is still difficult to maintain both structural stability and fast electrochemical kinetics after prolonged charge–discharge operation, especially at high current densities. In many MnO-based electrodes, repeated conversion reactions usually lead to particle aggregation, unstable interfaces, and gradual capacity decay. Therefore, improving the structural durability and charge-transfer behavior of MnO electrodes remains an important issue.

In this work, interconnected MnO nanorods coated with a nitrogen-containing carbon layer were prepared through hydrothermal synthesis, dopamine self-polymerization, and subsequent annealing treatment. Different from conventional MnO/C particles, the present material maintains a one-dimensional rod-like structure after carbonization, while the carbon shell remains uniformly attached to the surface of the nanorods. Some nanorod ends remain partially open after coating, which facilitates electrolyte infiltration and accelerates Li^+^ diffusion within the electrode. In addition, the nitrogen-doped carbon layer enhances electron transport within the electrode and mitigates the mechanical degradation associated with repeated structural fluctuations during electrochemical cycling.

According to the electrochemical test results, a reversible capacity of 912.9 mA h g^−1^ was delivered by the electrode under a current load of 0.1 A g^−1^; and after 1400 cycles at 5.0 A g^−1^, the electrode still delivers a reversible capacity of 289.6 mA h g^−1^, this value is about 114.2% of the original amount, so it can be concluded that the capacity shows a considerable improvement after activation. Moreover, CV, EIS, capacitive contribution analysis, and GITT results further indicate that the combined effect of the nanorod structure and N-doped carbon coating can effectively improve charge-transfer kinetics and Li^+^ diffusion behavior. This strategy put forward here is generalizable to many other conversion-type transition-metal-based oxide anodes and can serve to promote the development of cost-effective, environmentally friendly, stable batteries, *etc.*

## Results and discussion

### Characterization

**Fig. 1:**
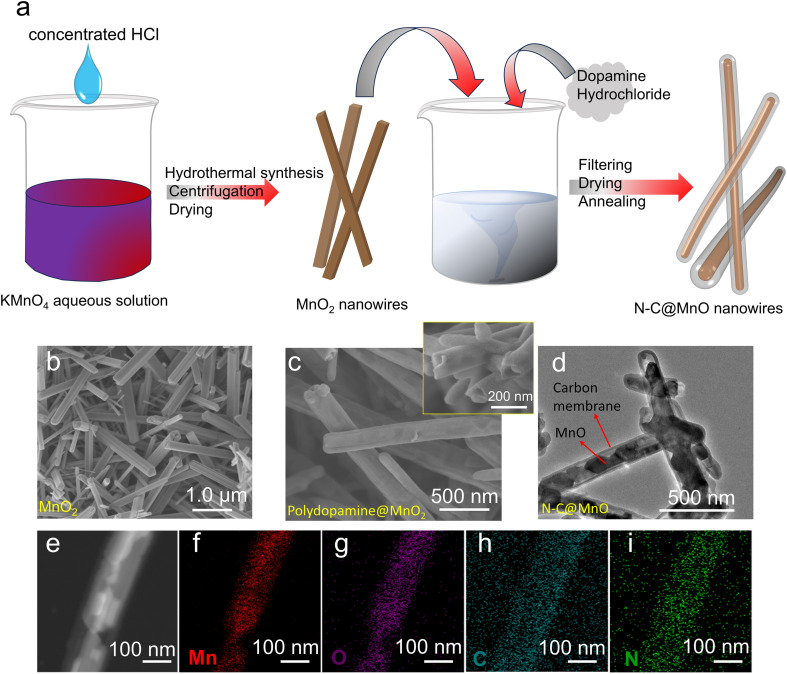
(a) Overview of the N–C@MnO nanorod preparation process. (b) SEM micrograph of MnO_2_ nanorods. (c) SEM micrograph of polydopamine@MnO_2_ nanorods, and the illustration in the top – right section shows that some of the nanorods in the wrapping are in an open-ending state. (d) TEM image of N–C@MnO nanorods; (e–i) TEM micrograph and respective elemental mapping images of N–C@MnO.

The preparation procedure of N–C@MnO is illustrated in Fig. 1a. First, according to the above protocol,^18^ MnO_2_ nanorods were synthesized by single-pot hydrothermal reaction; after several times of centrifugation and washing with deionized water, the dark precipitate obtained was subjected to air drying at 60 °C, yielding MnO_2_ with a mass yield of 79%. In the second step, a polydopamine membrane was prepared on the surface of MnO_2_ nanorods by stirring a mixture of MnO_2_ nanorods and dopamine hydrochloride in ethanol and purified water at room temperature. Finish coating, then filter and wash the material to obtain a clean sample; subsequently, the obtained sample was dried at 60 °C under oven conditions until a constant weight was achieved. Lastly, raise the temperature of the polydopamine-coated precursor in argon gas to 750 °C and keep it there for another 2.5 hours. At the same time, thermal treatment can change the polydopamine layer into nitrogen-containing carbon film and promote topotactic reduction of MnO_2_ to MnO; without the carbon sheath, there would be uncontrolled crystal growth and the formation of large, agglomerated particles.

SEM and TEM were used to carry out morphological characterisation. Fig. 1b shows that the hydrothermally prepared MnO_2_ are in a good one-dimensional rod shape with uniform dimensions. After the addition of polydopamine (Fig. 1c), most of the rods have been completely covered by a polymer layer; however, part of the rod tips shown in the inset are still open and lack this coating, which is a typical phenomenon in surface-coating experiments. After annealing (Fig. 1d), there are rough spots and pores in the interior of the nanorod due to gas generation and recrystallisation during the reduction of MnO_2_ to MnO, but the outer nitrogen-containing carbon film that originates from polydopamine is still continuous and follows the shape of the rod. EDS element maps (Fig. 1e–i) confirm the successful incorporation of Mn, O, C, and N species with a homogeneous spatial distribution throughout composite architecture; thus, it can be verified that N–C@MnO has been successfully formed. Through the TEM characterization, the thickness of the carbon layer encapsulating manganese oxide in N–C was measured to be mainly among 20–30 nm, according to the result presented in Fig. S1a. The thermal decomposition behavior of the sample to estimate the carbon content was evaluated through thermogravimetric testing (Fig. S1b).^19^ An initial mass loss of approximately 8–10% below 280 °C was detected, which mainly resulted from the release of adsorbed moisture and decomposition of residual surface species. Therefore, this low-temperature weight variation was excluded from the carbon calculation. A pronounced mass loss was detected in the temperature range of 280–385 °C, which can be assigned to the oxidative decomposition of the carbon coating. Considering that MnO is oxidized to Mn_3_O_4_ (confirmed by XRD in Fig. S2) during heating in air,^19^ the final residue obtained at 800 °C was corrected according to the MnO/Mn_3_O_4_ mass conversion relationship. The N–C@MnO composite was found to contain approximately 35 wt% carbon after correction. The appropriate carbon fraction provides a conductive network while maintaining a high proportion of electrochemically active MnO, which contributes to enhanced rate performance and improved cycling stability of the electrode.

**Fig. 2:**
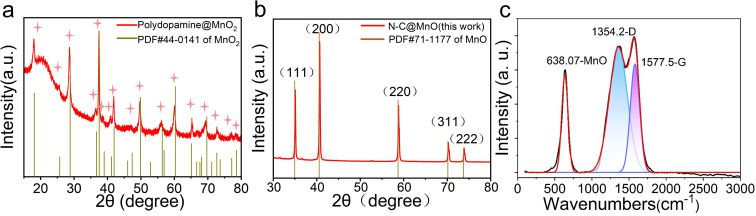
XRD patterns of polydopamine@MnO_2_ in (a) and N–C@MnO in (b), respectively. (c) Raman spectrum of N–C@MnO sample.

Powder X-ray diffraction was employed to elucidate the crystalline phases and the reference patterns of tetragonal MnO_2_ (JCPDS 44-0141) and rock-salt MnO (JCPDS 71-1177) were compared with this. The diffractogram of the as-synthesised precursor (Fig. 2a) has peaks marked by cross-star symbol, which are consistent with the tetragonal MnO_2_ phase, and it is phase-pure before coating. After the pyrolysis step, all the diffraction peaks have shifted to the positions of JCPDS 71-1177 (Fig. 2b), and there is no discernible residual oxide impurity; thus, MnO_2_ has been quantitatively converted to MnO.^9^

The characteristic Raman features of N–C@MnO are given in Fig. 2c. The peak at 638.07 cm^−1^ is associated with Mn–O lattice vibrations in MnO, confirming the successful formation of the MnO phase. Two characteristic Raman bands located at 1354.2 and 1577.5 cm^−1^ can be identified as the D band associated with disordered carbon domains and the G band originating from graphitic sp^2^ carbon networks, respectively. The obtained *I*_D_/*I*_G_ ratio of 1.07 indicates a defect-rich carbon structure with partial graphitization. The defective carbon framework not only enriches electrochemically accessible sites but also shortens charge transport pathways, ultimately improving the overall performance of N–C@MnO.^20^

**Fig. 3:**
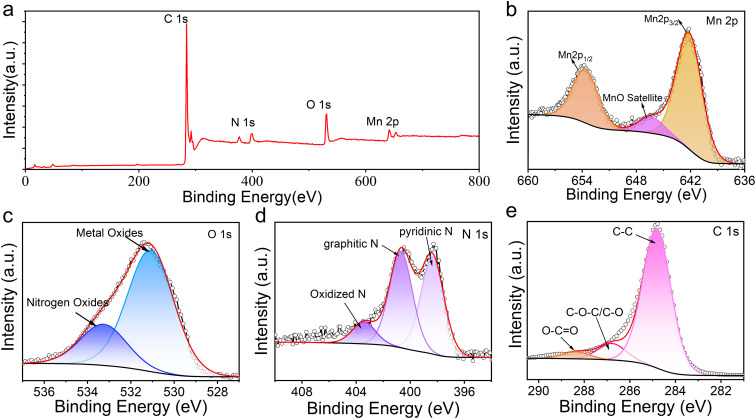
(a) XPS survey spectrum and high-resolution deconvoluted with peak fitting analysis of Mn 2p, O 1s, N 1s, and C 1s in (b–e) for the N–C@MnO sample.

The surface chemical composition and bonding configurations of N–C@MnO were analyzed by XPS. The four main ranges of the full survey scan (Fig. 3a) are Mn 2p, O 1s, C 1s and N 1s, and all the expected constituent elements have been verified. As shown in the fit of the Mn 2p region (Fig. 3b), at 642.3 eV there is a spin–orbit split component for Mn 2p_3/2_ and at 653.7 eV there is a spin–orbit split component for Mn 2p_1/2_, and together they can observe a shake-up satellite around 646.5 eV; this is regarded as a spectroscopic fingerprint of the Mn^2+^ oxidation state in MnO.^5,13,21,22^ therefore, it can be concluded that the manganese oxide phase is MnO. The O 1s fitted peaks in Fig. 3c show two main components at 533.3 eV and 531.1 eV, originating from nitrogen oxides and metal oxides, respectively. Together with N 1s and C 1s signals, this also indicates that there is a carbonaceous outer membrane containing oxygen and nitrogen. Fig. 3d shows the peak deconvolution of the N 1s envelope, and there are three nitrogen species at 398.4 eV (pyridinic-N), 400.7 eV (graphitic-N) and 403.4 eV (oxidised-N); similarly, the C 1s spectrum (Fig. 3e) can be divided into a main graphitic C–C signal at 284.8 eV, flanked by C–O/C–O–C at 286.8 eV and O–C=O at 288.3 eV.^6,23^ The dominant sp^2^ C–C component suggests the generation of an aromatic carbon framework during the thermal conversion of the polydopamine precursor. Nevertheless, the existence of sp^2^-hybridized carbon alone cannot be regarded as direct evidence of complete graphitization. In combination with the Raman analysis (Fig. 2c), where an *I*_D_/*I*_G_ value of 1.07 reveals a relatively defective carbon structure, the carbon layer is more appropriately described as a partially graphitized carbon coating with abundant defects. Such a carbon structure can provide enhanced electronic conductivity while retaining sufficient defect sites for electrochemical reactions. Integrating the XRD, Raman and XPS analyses, the product is identified as N-doped carbon membrane-encapsulated MnO.

### Electrochemical analysis

**Fig. 4:**
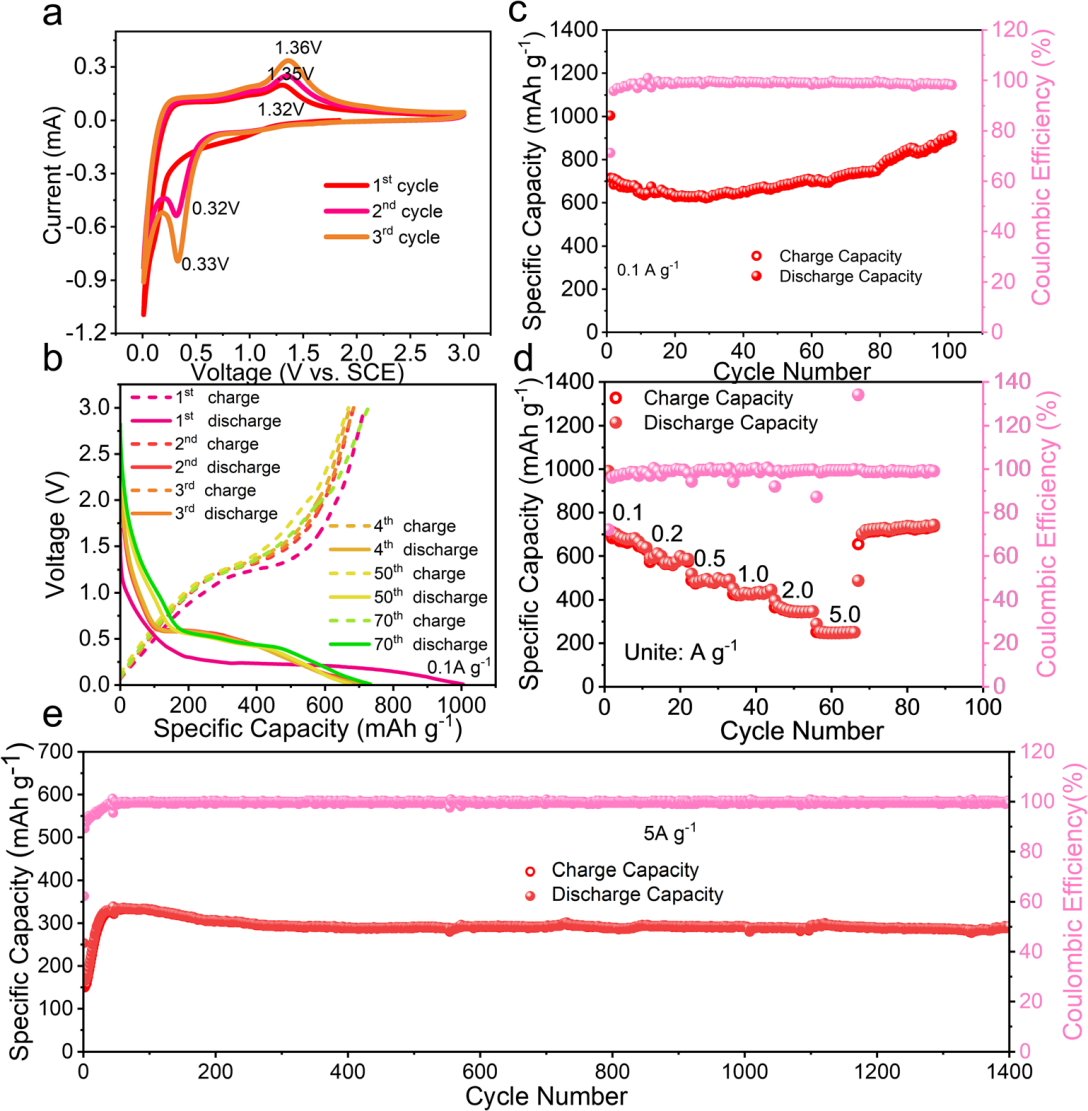
(a) First three CV cycles at scanning speed of 0.2 mV s^−1^. (b) GCD curves at the 1st, 2nd, 3rd, 4th, 50th, and 70th cycles. (c) Rate performance of N–C@MnO electrode form 0.1 to 5 A g^−1^. (d) Cyclic stability and coulombic efficiency at 0.1 A g^−1^ for 100 cycles. (e) Long-life cycling behaviour at 5 A g^−1^ for 1400 cycles.

To determine whether N–C@MnO is a suitable battery anode, it was added to Li/Li^+^ half-cell systems. A combination of several methods was employed for characterization to study both charge-storage capacity and reaction kinetics simultaneously; these included CV, GCD, EIS, capacitive contribution quantification and GITT. Cyclic voltammetry (Fig. 4a) was taken between 0.01–3.00 V *vs.* Li/Li^+^ with a scan rate of 0.2 mV s^−1^. Only one broad oxidation feature is present near 1.32 V in the first cycle; as there is no obvious cathodic counterpart, it can be concluded that irreversible parasitic reactions, such as SEI layer formation and electrode restructuring, are dominant and inhibit the reduction of bulk MnO in this initial cycle. From the second cycle, the CV curve has changed significantly; it is clear that oxidation and reduction peaks at around 1.35 V and close to 0.33 V have appeared, and the structure and interface are stable. After the initial cycle, a stable SEI layer is generated and subsequently maintained in a kinetically stable state; therefore, further parasitic lithium consumption has been suppressed. This in-situ-formed pre-activated state allowed for the full reversible conversion of MnO ↔ Mn + Li_2_O in the second cycle and beyond. Quantitatively, the ∼1.35 V oxidation peak corresponds to delithiation: Mn + Li_2_O→ MnO + 2Li^+^ + 2e^−^, and the ∼0.33 V reduction peak is to lithiation: MnO + 2Li^+^ + 2e^−^ → Mn + Li_2_O. A stable and well-defined separation between the oxidation and reduction peaks is observed in the second and third cycles, with a potential difference of approximately 1.02 V apart for 1.35 V and 0.33 V, and the peak current gradually increases during continuous cycling; thus, the manganese oxide anode exhibits excellent reversibility and good structural stability. The CV plots of the 2nd and 3rd cycles are similar; therefore, good reversibility of the electrochemical reaction has been verified.^24^

The GCD curves obtained at 0.1 A g^−1^ in the same voltage range are displayed in Fig. 4b, the first discharge trace has a large plateau at about 0.25 V, and then the potential drops to the cut-off of 0.01 V, which is consistent with a two-stage conversion of Mn^2+^ to Mn^0^. The GCD profiles at the 2nd, 3rd, 4th, 50th and 70th cycles are very close; thus, it can be concluded that the lithium storage kinetics have been highly reversible after the initial activation in the first cycle.

The charge–discharge characteristic of the N–C@MnO electrode at 0.1 A g^−1^ is shown in Fig. 4c. During the cycling process, the electrode experiences a slight capacity decline in the initial stage, which is subsequently accompanied by a continuous enhancement in reversible capacity. This behavior is likely associated with the progressive exposure of electrochemically accessible sites and the continuously enhanced electrode/electrolyte interaction during repeated lithiation/delithiation processes.^25^ As a result, the electrode achieves a reversible discharge capacity of 912.9 mA h g^−1^ at the 100th cycle The first-cycle coulombic efficiency of 71.3% is comparable to previously reported values for MnO-based conversion-type anodes.^26^ The relatively low initial efficiency originates from the irreversible Li^+^ loss occurring during the initial lithiation process, including the establishment of a stable SEI film and unavoidable parasitic reactions occurring at the electrode–electrolyte interface. Moreover, the defect-containing N-doped carbon coating can offer additional lithium-storage sites, which may lead to enhanced irreversible Li^+^ depletion in the initial lithiation cycle. Nevertheless, the carbon shell plays a crucial role in improving electron transport and maintaining structural integrity during repeated charge/discharge processes, thereby enabling the electrode to achieve favorable cycling stability.^27,28^ Strategies such as electrochemical prelithiation or optimization of the carbon coating thickness and surface characteristics could be explored to further enhance the initial coulombic efficiency.^29,30^ The coulombic efficiency rises to 95.9% in the 2nd cycle before reaching a stable range of 98–100% after the formation of an interfacial film. An initial discharge capacity of 1004.7 mA h g^−1^ is delivered by the N–C@MnO electrode, which is mainly attributed to the reversible MnO conversion reaction with Li^+^ as well as the additional lithium storage provided by the conductive carbon matrix.

The rate performance shown in Fig. 4d was evaluated at current densities from 0.1 to 5.0 A g^−1^. The discharge capacity of the electrode is 710.29 mA h g^−1^ at a rate of 0.1 A g^−1^, and it could maintain about 350.8 mA h g^−1^ under 2 A g^−1^ and 250.8 mA h g^−1^ under 5 A g^−1^, so it is suitable for fast-charging conditions. Upon return to 0.1 A g^−1^, the capacity reached 740.5 mA h g^−1^, which was higher than the initial baseline and showed that the electrode architecture was still in good condition.

Furthermore, as shown in Fig. 4e, under a high-current condition of 5.0 A g^−1^, an obvious activation process occurs in the first 45 cycles, during which the capacity rises rapidly, followed by a short plateau and then stabilization. Such behavior suggests that the active material is not fully utilized at the beginning of cycling and undergoes a progressive activation process. During repeated lithiation and delithiation, the MnO phase can be gradually transformed into finer electrochemically active domains, which facilitates electrolyte penetration and shortens the lithium-ion diffusion distance.^31,32^ Meanwhile, continuous cycling improves the accessibility of the N-doped carbon shell, allowing more defect sites and nitrogen-containing functionalities to participate in lithium storage.^33^ The intimate contact between MnO and the conductive carbon framework also contributes to enhanced charge transport and more efficient utilization of the active material.^9^ Accordingly, the reversible capacity gradually recovers in the initial cycles and maintains a stable trend during subsequent long-term cycling, reflecting the effective structural preservation and favorable electrochemical reversibility of the N–C@MnO electrode, retaining 289.6 mA h g^−1^ after 1400 cycles, with a capacity retention of 114.2% calculated from the final-to-initial capacity ratio. Many other papers have also reported this incremental capacity recovery trend for transition metal oxide anodes.^34–36^

For comparison, the electrochemical behaviors of pristine MnO (XRD in Fig. S2) were also investigated and the corresponding CV curves together with GCD behavior, rate performance, and cycling stability are presented in Fig. S3 and S4. When compared with the N–C@MnO electrode, pristine MnO exhibits relatively weaker electrochemical reversibility and lower capacity during the rate cycling. In addition, the degraded rate performance of MnO indicates insufficient charge-transfer kinetics and limited structural adaptability during repeated lithiation/delithiation processes. These results further confirm the crucial contribution of the nitrogen-doped carbon shell to enhanced reaction kinetics and improved electrode stability.

A comparison with recently reported MnO-based anodes is presented in Table S1 to evaluate the electrochemical performance of the N–C@MnO electrode.^1,2,12,13,37–41^ Although several MnO-based composites reported in recent years deliver comparable or even higher reversible capacities at relatively low current densities, maintaining stable capacity during high-rate cycling remains challenging. In comparison, the N–C@MnO electrode retains 289.6 mA h g^−1^ even after 1400 cycles at 5 A g^−1^, demonstrating excellent long-term lithium storage capability under fast charge/discharge conditions. This enhanced durability stems from the special structural configuration of N–C@MnO. The N-doped carbon shell provides a confined nanoscale environment that stabilizes the MnO active phase and limits particle migration and crystal coarsening during repeated lithiation/delithiation processes. Consequently, the active domains generated during the conversion reaction are maintained at a relatively small scale, facilitating continuous reversible lithium storage and sustaining stable electrochemical performance during prolonged cycling.

**Fig. 5:**
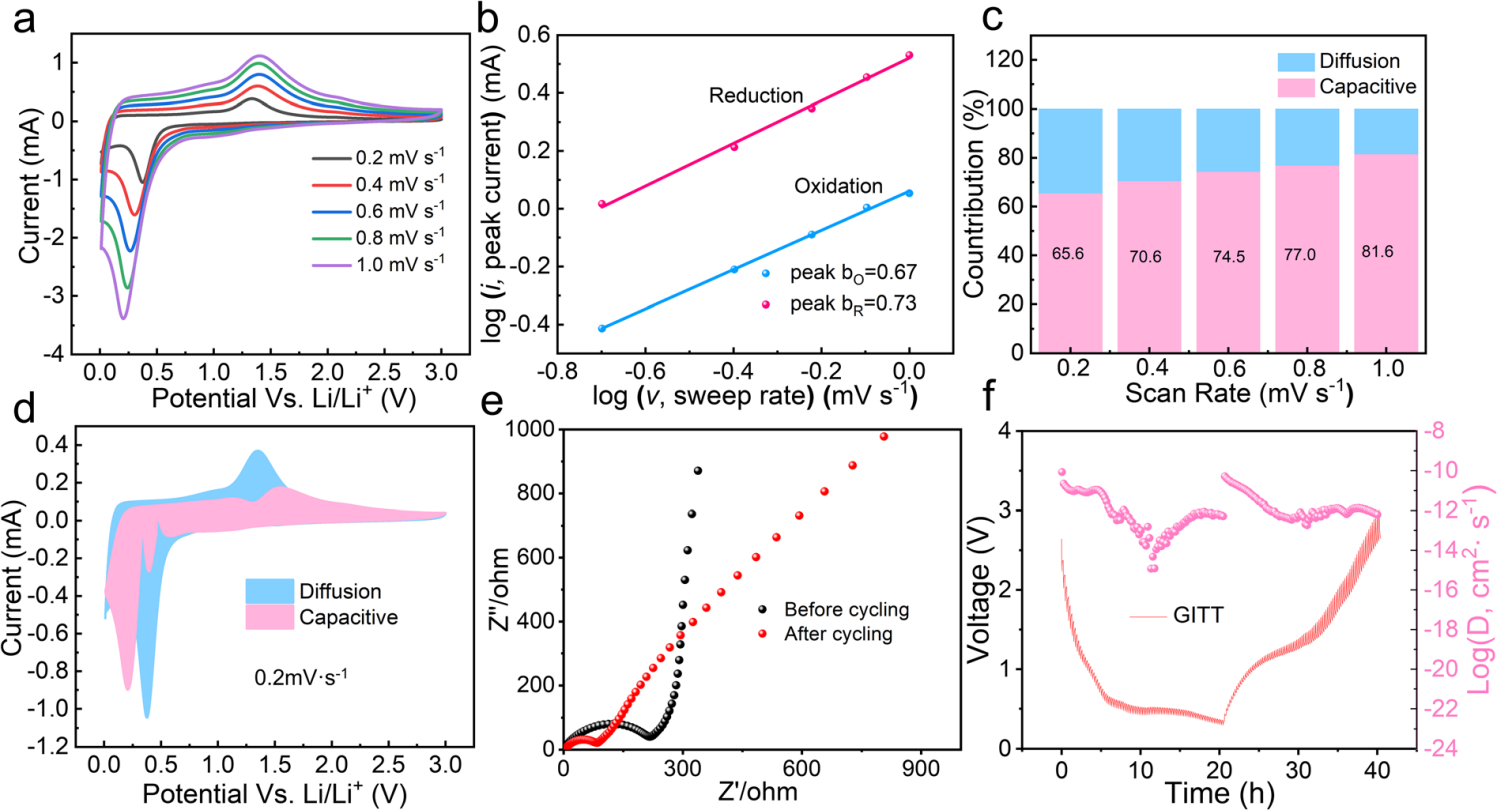
(a) Scan-rate-dependent cyclic voltammetry behaviors of the N–C@MnO electrode within the range of 0.2–1.0 mV s^−1^. (b) Calculation of the ‘*b*’ value employing the peak current–scan rate relationship formula (2). (c) Capacitive and diffusion contributions at varying scan rates. (d) CV curve recorded at 0.2 mV s^−1^, with the capacitive contribution marked in the pink area. (e) Nyquist plots before and after the first cycle. (f) GITT profile and the corresponding Li^+^ diffusion coefficients (*D*_Li^+^_).

To decipher the electrochemical kinetic characteristics, CV measurements was conducted at sweep rates spanning 0.2–1.0 mV s^−1^, as presented in Fig. 5a. The kinetic mechanisms were analyzed using the eqn (1) and (2):

1 *i* = *a* × *v*^*b*^

2 log(*i*) = *b* × log(*v*) + log(*a*)

‘*a*’ and ‘*b*’ are the empirical fitting parameters in the above expressions, and ‘*b*’ is a mechanism index for a power law; that is, if *b* ≈ 0.5, it shows diffusion-limited (bulk intercalation) behaviour, and if *b* ≈ 1.0, it is purely capacitive (surface-confined) charge storage. The anodic and cathodic *b* values extracted for N–C@MnO—0.67 and 0.73 (Fig. 5b) are in the middle; thus, it can be determined that the lithium storage mechanism is a hybrid type mainly controlled by diffusion.^9^

3 *i* = *k*_1_*v* + *k*_2_*v*^1/2^

4
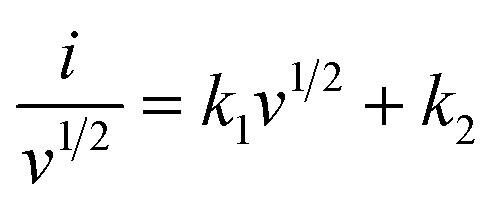


By plotting *v*^1/2^ against *i*/*v*^1/2^, the surface capacitive contributions (*k*_1_) were decoupled from the diffusion-limited processes (*k*_2_). The capacitive contribution (*k*_1_) shows a clear increase with increasing scan rates, reaching 81.6% from an initial value of 65.6% over the range of 0.2–1.0 mV s^−1^ (Fig. 5c), and the influence of surface reactions at higher scan rates is thus more prominent. At 0.2 mV s^−1^ specifically (Fig. 5d), the shaded area of capacitive storage already makes up 65.6% of the total stored charge.

EIS Nyquist plots (Fig. 5e) of the electrode prior to cycling and after the initial galvanostatic charge–discharge process were obtained to see if there were any changes in interfacial resistance. All the spectra exhibit a high-frequency depressed arc associated with interfacial charge-transfer processes, followed by an inclined Warburg region at low frequencies related to Li^+^ diffusion within the electrode material. The initial charge-transfer resistance is about 217.2 Ω, and after the first cycle, it has decreased to about 83.4 Ω; at this time, the electrodes will start to work together and form a good conductive interface with the SEI film and active material. These changes collectively accelerate interfacial charge transfer and sustain high reaction efficiency throughout subsequent cycles.^15^

GITT experiments were conducted at 0.1C to explore the ion-transport characteristics of N–C@MnO across different states of charge more deeply, and *D*_Li+_ values at each electrochemical stage were obtained using eqn (5) and plotted in Fig. 5f:5
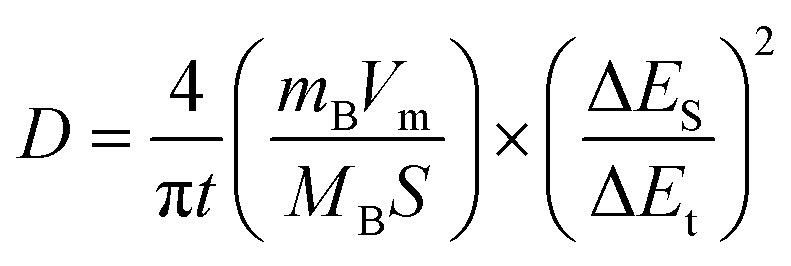


GITT shows that the average Li^+^ diffusion coefficient in discharge is 3.90 × 10^−12^ cm^2^ s^−1^, and in charge it is 4.69 × 10^−12^ cm^2^ s^−1^. The *D*_Li^+^_ profile is not uniform in the voltage range; it is relatively high at the beginning of discharge due to surface adsorption, but with an increase in lithiation, bulk lattice insertion becomes the primary mechanism and thus *D*_Li^+^_ gradually decreases. There is a small minimum in *D*_Li^+^_ around the middle of the discharge plateau; this is due to the combined effect of a reversible redox reaction, defect-site trapping, and both surface and bulk effects.

To further investigate how carbon coating affects the electrochemical kinetic behavior, the kinetic behaviors of pristine MnO were analyzed by scan-rate-dependent CV, EIS, and GITT measurements (Fig. S5). The corresponding results reveal that pristine MnO possesses slower charge-transfer kinetics compared with N–C@MnO. The enhanced kinetic response of N–C@MnO is associated with the conductive nitrogen-doped carbon framework, which provides continuous electron-transfer pathways and facilitates ion transport within the interfacial region between the electrode and electrolyte. The detailed kinetic analysis of pristine MnO is presented in the SI.

Above all, the mechanism for the good electrochemical performance of N–C@MnO is the combination of the three above structures: N-containing carbon can form a good electronic conductor network; the flexible covering material can withstand swelling and shrinkage during the course of cycling without breaking; and the interconnected array of nanorods is favourable for electrolyte diffusion and reduces the path length of ion transport. The above parts will present the kinetics and mechanical stability of the material that can be employed in high-capacity future high-performance Li-ion energy storage devices.

## Experimental

### Preparation of the manganese dioxide (MnO) precursor—MnO_2_

MnO_2_ nanorods were synthesized hydrothermally according to the protocol in ref. 18. Briefly speaking, add 0.395 g of KMnO_4_ to 45 mL of deionised water, stir with a magnet stirrer, and then slowly add 0.6 mL of concentrated HCl (12 M) dropwise to the solution. A portion of the dark-purple solution was transferred into a polytetrafluoroethylene (PTFE)-lined reactor, followed by hydrothermal treatment at 160 °C for 10 h. After passive cooling to room temperature, centrifuge the black precipitate to obtain it; then, washed repeatedly using deionized water up to the pH of the wash supernatant is neutral, and finally dry it overnight at 60 °C in the air.

### Preparation of N–C@MnO

For polydopamine coating of the prepared MnO_2_ nanorods, 0.087 g of MnO_2_ nanorods was mixed with 0.189 g dopamine hydrochloride in 200 mL of an ethanol/water solvent system (1 : 2, v/v). Ammonium hydroxide solution (about 0.2 mL) was then added dropwise to raise the pH to approximately 8, and for 12 hours, a stir was carried out at room temperature to ensure uniform dispersion of the polymer. Filter the Polydopamine-coated product, wash with ethanol, and then dry at 60 °C for an extended period. Subsequently, the precursor was thermally treated under N_2_ protection with a heating rate of 18 °C min^−1^, and the temperature was maintained at 700 °C for 2.5 h. This process simultaneously generated the nitrogen-containing carbon layer and converted MnO_2_ into MnO, yielding the final N–C@MnO composite.

### Characterization of the materials

SEM (SU8220) and TEM (JEM-2100F, 200 keV) were used to examine the shape and internal structure of the powder, and EDS in TEM mode was employed to obtain elemental distribution maps. Crystal structure was obtained by XRD (D/max 2500pc, Cu Kα radiation), and surface elemental states were examined using XPS (Thermo ESCALAB 250Xi with an Al Kα source). The carbon fraction in the N–C@MnO composite was determined by thermogravimetric measurement using a METTLER TOLEDO instrument. The sample was heated in air from ambient temperature up to 800 °C during the analysis.

### Electrochemical measurements

Electrodes were fabricated by blade coating. Briefly, N–C@MnO powder, Super P conductive carbon, and PVDF binder were mixed in a mass proportion of 7 : 2 : 1 and homogenized in NMP to form a uniform slurry. The resulting slurry was deposited onto copper foil substrates and dried under vacuum at 80 °C for 10 h. The dried electrode film was subsequently punched into circular discs with a diameter of 12 mm. CR2032 coin-type half-cells were assembled in an Ar-protected glovebox, using lithium foil as the counter/reference electrode. The electrolyte consisted of 1 M LiPF_6_ dissolved in an EC/DMC solvent mixture (1 : 1, v/v). Electrochemical measurements, including galvanostatic charge–discharge, rate capability evaluation, CV, and EIS tests, were performed within 0.01–3.00 V using a NEWARE CT-3008A battery testing system and a CHI 760E workstation.

## Conclusion

In summary, N–C@MnO nanorods with a nitrogen-doped carbon coating were successfully fabricated through a combination of hydrothermal growth, dopamine-derived polymer coating, and subsequent carbonization treatment. Rather than simply introducing a carbon layer onto MnO, this work constructs an integrated one-dimensional MnO/carbon hybrid architecture, where the conductive carbon coating is tightly coupled with the MnO nanorods to form a continuous electron transport network. The interconnected nanorod framework provides shortened pathways for Li^+^ migration and facilitates electrolyte penetration, while the flexible carbon shell effectively accommodates the mechanical strain induced by repeated structural expansion and contraction associated with repeated Li^+^ insertion and extraction. Owing to the aforementioned structural characteristics, the N–C@MnO electrode delivers improved reversible capacity, enhanced rate capability, and stable long-term cycling performance, retaining a reversible capacity of 289.6 mA h g^−1^ after 1400 cycles at 5 A g^−1^. Furthermore, electrochemical kinetic investigations reveal that the synergistic interaction between the MnO nanorods and N-doped carbon coating contributes to accelerated interfacial charge transfer and optimized Li^+^ transport behavior. This work establishes a feasible strategy for engineering carbon-modified MnO nanostructures and highlights its potential for developing stable conversion anodes in lithium-ion batteries.

## Author contributions

Q. Y. Y. responsible for the entire experimental process, data analysis, graphing, and manuscript writing. J. Q. W. contributed to investigation, formal analysis, and data curation. Y. M. L. and C. J. L. contributed to investigation and data curation. X. Y. F. and N. W. contributed to investigation and original draft preparation. M. L. G. contributed to original draft preparation. All authors reviewed the manuscript, made critical revisions, and approved the final version.

## Conflicts of interest

All contributing authors confirm the absence of any competing financial interests.

## Supplementary Material

RA-OLF-D6RA03883B-s001

## Data Availability

All data needed to support the conclusions in the paper are presented in the manuscript and the supplementary information (SI). Additional data related to this paper may be requested from the corresponding author upon request. Supplementary information is available. See DOI: https://doi.org/10.1039/d6ra03883b.
