# Supplementary material for: Nitrogen-containing carbon membrane-encapsulated MnO nanorods for ultrastable lithium-ion batteries
Source: RSC Adv. 2026 Jul 13. Online ahead of print. doi: 10.1039/d6ra03883b (PMC13358705; doi:10.1039/d6ra03883b)
Supplement: RA-OLF-D6RA03883B-s001 [file RA-OLF-D6RA03883B-s001.pdf]

Supplementary Information (SI) for RSC Advances.
This journal is © The Royal Society of Chemistry 2026

Electronic Supplementary Information Material

Nitrogen-containing Carbon Membrane-Encapsulated MnO Nanorods for Ultrastable Lithium-Ion Batteries

QiuYun Yang*, Jiaqiang Wei, Yumu Liu, Xiaoyu Fan, Chunjian Liu*, Na Wang*, and Minglei Guo*

1 Institute of Electrical and Electronic Engineering, Anhui Science and Technology University, Bengbu, Anhui 233000, China

* Corresponding author

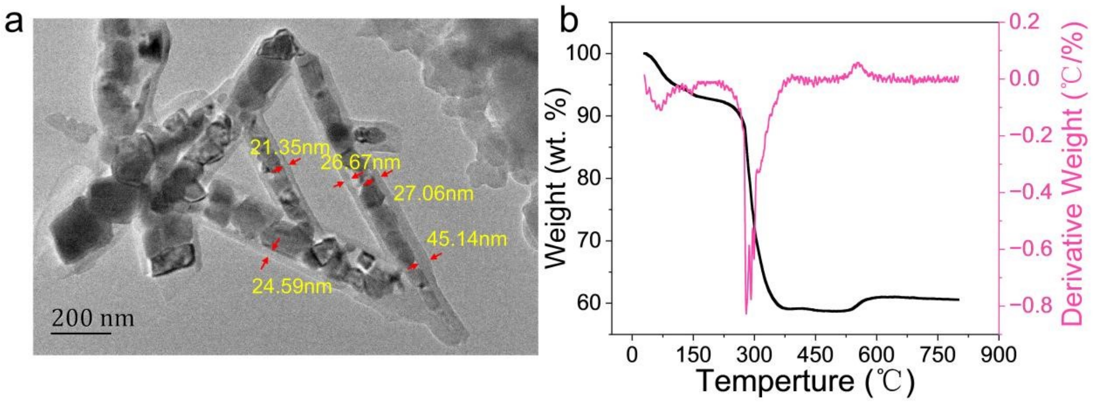

Figure S1 (a) TEM image of N-C@MnO for the thickness of the carbon shell; (b) TGA curve of N-C@MnO.

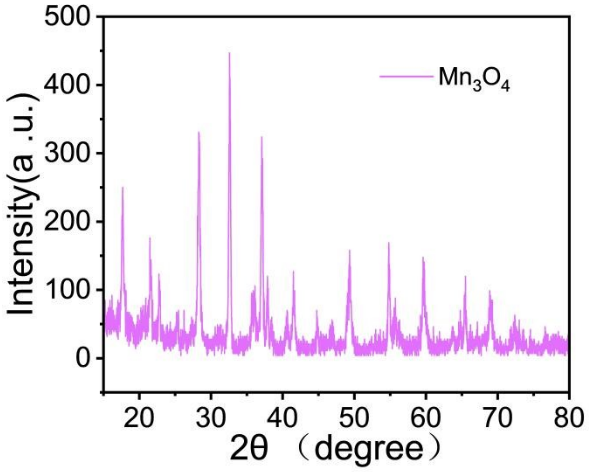

Figure S2 XRD pattern of Manganese oxide after TGA test.

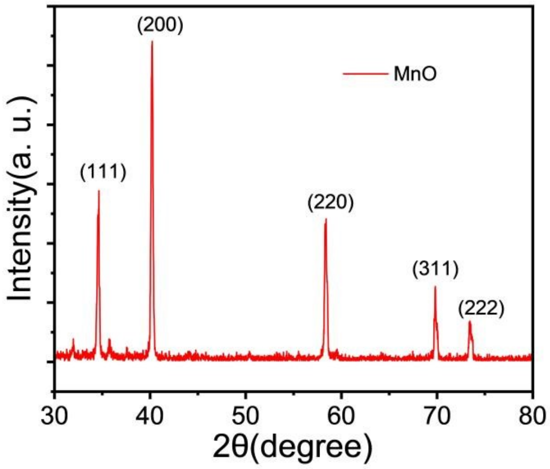

Figure S3 XRD pattern of prepared MnO.

Preparation and structural characterization of MnO:

The obtained MnO_2 nanorods were further treated at 1100 °C under a N_2 atmosphere for 2.5 h to induce the reduction of manganese oxide into MnO. During this high-temperature treatment, significant grain growth occurred, resulting in relatively large MnO particles. The obtained MnO powders were subsequently ground before electrode preparation to ensure uniform mixing with other electrode components.

The phase structure of the prepared MnO sample was investigated by X-ray diffraction (XRD). As shown in Figure S3, all diffraction peaks can be indexed to the cubic MnO phase, with the characteristic reflections corresponding to the (111), (200), (220), (311), and (222) planes. No diffraction peaks associated with other manganese oxide species are observed, indicating the formation of phase-pure MnO. The XRD pattern is consistent with the MnO diffraction peaks shown in Figure 2b, further verifying the successful conversion of the manganese oxide precursor into MnO after thermal treatment.

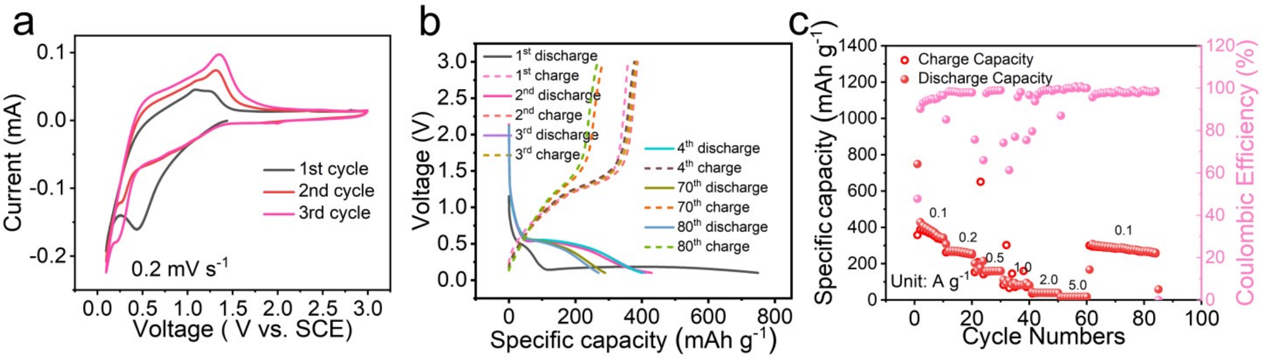

Figure S4 (a) First three CV cycles at scanning speed of 0.2 mV s^{-1} of MnO. (b) GCD curves of MnO at the 1st, 2nd, 3rd, 4th, 70th, and 80th cycles. (c) Rate performance of MnO electrode form 0.1 to 5 A g^{-1} .

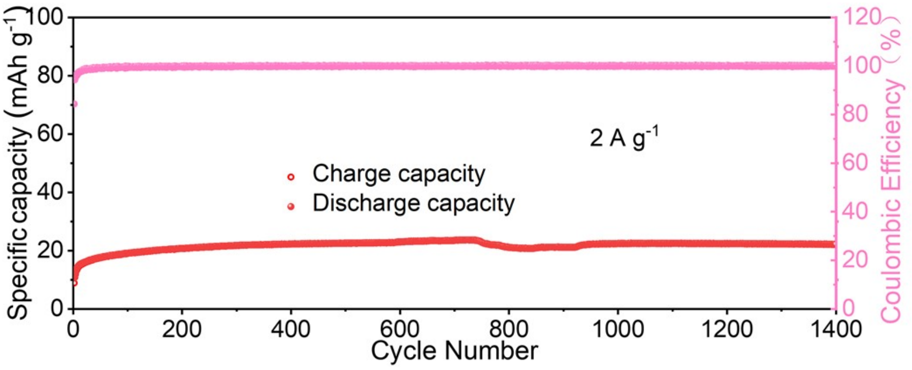

Figure S5 Cyclic stability and Coulombic efficiencyat 2 A g^{-1} for 1500 cycles.

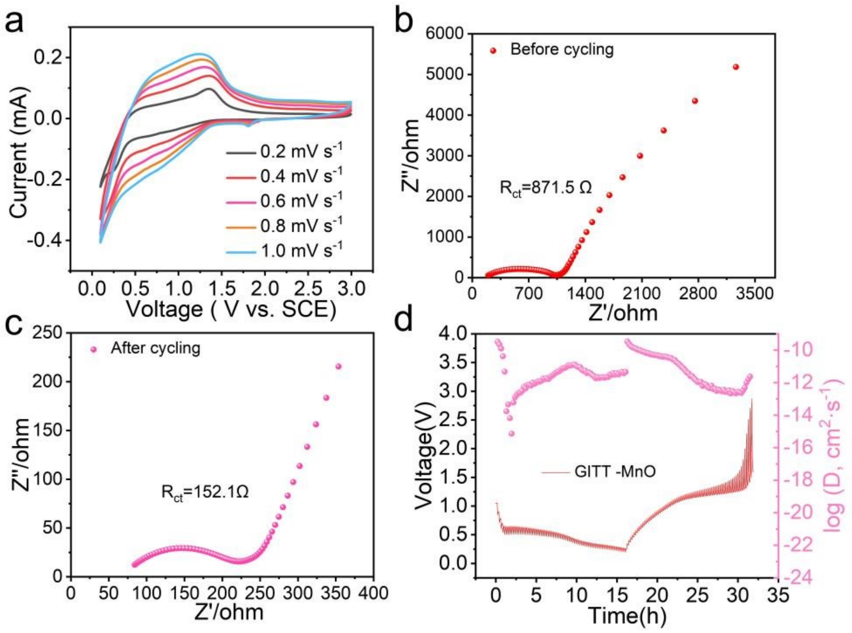

Figure S6 (a) CV cycles of MnO at potential sweep rates between 0.2 and 1.0 mV s^{-1}. Nyquist plotsof MnO electrode (b) before cycling and (c) after the first cycle; (d) GITT profile and the corresponding Li^+ diffusion coefficients (D_{Li^+}) of MnO electrode.

Figure S6a shows the CV curves of MnO manganese at different scan rates. Compared with N-C@MnO, the MnO electrode exhibits much weaker redox peak currents, indicating sluggish electrochemical reaction kinetics and reduced reversible reaction activity. Figure S6b shows the EIS spectrum of the MnO electrode before the cycling process(R_{ct} - 871.5Ω), and Figure S6c presents the EIS spectrum after 1st cycling(Rct - 152.1Ω). The large decrease in R_{ct} after the first cycle suggests an activation process of the MnO electrode. Nevertheless, compared with bare MnO, the conductive carbon coating in N-C@MnO provides a continuous electron transport pathway and effectively lowers the charge-transfer resistance. The GITT curve at 0.1C of the MnO electrode is presented in Figure S6d. As demonstrated in Figure 4f, the N-C@MnO electrode exhibits improved Li^+ transport behavior compared with pristine MnO. However, the calculated Li^+ diffusion coefficients of MnO is 1.30×10^{-11} of discharge and 3.45×10^{-11} of charge). This result can be explained by considering the role of the carbon layer and the reaction mechanism of MnO. The carbon coating mainly facilitates electron conduction, maintains structural integrity, and improves interfacial reaction kinetics, whereas Li^+ diffusion in MnO is largely determined by the intrinsic conversion reaction involving MnO and Li-containing phases. Moreover, the different active material loading amounts of the MnO electrode (~1.64 mg) and N-C@MnO electrode (~0.637 mg) may introduce additional variations during the GITT calculation.

Table S1. Comparison of specific capacity and cycling performance of recently reported MnO-based anodes for lithium-ion batteries.

**Electrode Material**	**Capacity (mAh g^{-1})**	**Current Density (A g^{-1})**	**Cycle Number**	**Ref.**
MnO@N-C	560	1.0	300	Ref.38
N–doped carbon encapsulated Mn_2O_3/MnO	683.6	0.5	1000	Ref.2
Porous MnO/pitch carbon composite	250	1.0	450	Ref.1
macro-porous MnO@CNT	872.87	1.0	750	Ref.39
MnO/Nitrogen-Doped Carbon	702	2.0	1000	Ref.40
Aminated Graphite-Reinforced MnO	705.942	1.0	1000	Ref.41
MnO@C/RGO composite	412	3.0	500	Ref.13
MnO NCs@rGO	1220	0.5	1000	Ref.42
PPy/MnO/C	918	0.1	120	Ref.12
**N-C@MnO (This work)**	**912.9**	**0.1**	**100**	**This work**
**N-C@MnO (This work)**	**289.6**	**5.0**	**1400**	**This work**
